# Benchmarking the paediatric T‐cell ALL subtype classifier, TALLSorts

**DOI:** 10.1111/bjh.70263

**Published:** 2025-12-12

**Authors:** Ozcan Gulbey, Terena James, Ruth E. Cranston, Dagmara Furmanczyk, Claire Schwab, Anna Lawson, Pam Kearns, Ajay Vora, Juliette Roels, Pieter Van Vlierberghe, Christine J. Harrison, Mark T. Ross, Amir Enshaei, Frederik W. van Delft, Anthony V. Moorman

**Affiliations:** ^1^ Leukaemia Research Cytogenomics Group, Centre for Cancer, Translational and Clinical Research Institute Newcastle University Newcastle upon Tyne UK; ^2^ Illumina Cambridge Ltd. Cambridge UK; ^3^ Cancer Research UK Clinical Trials Unit, School of Medical Sciences, College of Medicine and Health University of Birmingham Birmingham UK; ^4^ Department of Haematology Great Ormond Street Hospital London UK; ^5^ Department of Biomolecular Medicine Ghent University Ghent Belgium

**Keywords:** ALL, classifications, gene expression, T‐cell ALL

To the Editor,

T‐cell acute lymphoblastic leukaemia (T‐ALL) is a heterogeneous disease comprising 15 genomic subtypes.^1^ Although the outcome of children with T‐ALL has improved over the past few decades, survival rates still lag behind B‐cell precursor patients, and the outlook after relapse is dismal.^2^ Despite numerous studies, no robust validated genetic biomarkers have emerged that are used prospectively to tailor therapy. The rarity of the disease (15% of ALL) coupled with the large number of subtypes makes the identification and validation of T‐ALL biomarkers challenging. Furthermore, the different types of abnormality and subsequent variation in detection methods add further complexity. Even though there is an urgent need for robust biomarkers in T‐ALL, few studies have externally validated and evaluated potential biomarkers.^3^ A recent publication described a gene expression classifier (TALLSorts) capable of identifying seven distinct subtypes of T‐ALL.^4^ In this study, we classified 126 patients using TALLSorts, examined the subtypes using orthogonal genetic data and evaluated the clinical relevance of the subtypes.

Patients diagnosed with T‐ALL by standard morphological and immunophenotypic methods were treated on UKALL2003 (*n* = 87) or UKALL2011 (*n* = 39), approved by the Scottish Multi‐Centre or North Thames Research Ethics Committees.^5^, ^6^ Cytogenetics, fluorescence in situ hybridisation (FISH) and Multiplex Ligation‐dependent Probe Amplification (MLPA) data were generated, curated and coded as previously described.^3^, ^7^ Libraries were prepared using Illumina RNA preparation kits, and sequencing was performed on a NextSeq500 or NovaSeq 6000 system (see Supporting Information S1). FASTQ files were checked for quality control with FastQC^8^ (v.0.12.1) and MultiQC^9^ (v.1.0.dev0). Adapters and poor quality reads were trimmed with BBmap^10^ (v.39.06) and HOMER^11^ (v.4.11.1). Salmon^12^ (v.1.9.0) was used to align reads to hg38 reference genome index for estimated read counts from trimmed FASTQ files. Quant files from Salmon were imported into R (v.4.3.2) with tximport^13^ (v.1.30.0) to create a count matrix file. The TALLSorts algorithm was downloaded from the publicly available repository, GitHub, and installed as instructed.^4^ The count matrix file was run through the TALLSorts algorithm within the Miniconda environment in Ubuntu (v.20.04.6), producing predicted scores and subtypes for all 126 samples. In addition, gene fusions were identified using Arriba (v1.2.0).^14^ Briefly, trimmed FASTQ reads were aligned to hg38 and Gencode 28 using STAR (2.7.0e)^15^ before running Arriba fusion detection. We used standard statistical tests to compare subgroups and perform survival analysis (see Supporting Information S1).

The patient characteristics and outcomes of the cases included in this study were broadly representative of the total T‐ALL cohort, but the tested cohort was younger (Tables S1 and S2). The TALLSorts algorithm produces probability scores (0–1.0) indicating the likelihood the sample belongs to each subtype. We used the same threshold (0.5) as the original study to assign samples to a subtype. The probability scores for each subtype revealed that some distributions were discrete (e.g. *TLX1*, *TLX3*), while others were diffuse (e.g. *HOXA_MLLT10*) (Figure 1A). This finding contrasts with the original study, which reported discrete distributions for all subtypes.^4^ Across the 126 samples, there were 208 positive calls (i.e. probability score >0.5), ranging from 0 to 6 per case (Figure 1B). Among the 67 cases with >1 positive call, 64 (96%) cases included a *HOXA_MLLT10* call with the vast majority (53/64, 83%) being a secondary call (i.e. having the lower of the probability scores for that sample) (Figure S1). These observations suggest that the signature for the *HOXA_MLLT10* subtype is less robust than for other subtypes, consistent with the diffuse distribution observed.

**FIGURE 1:**
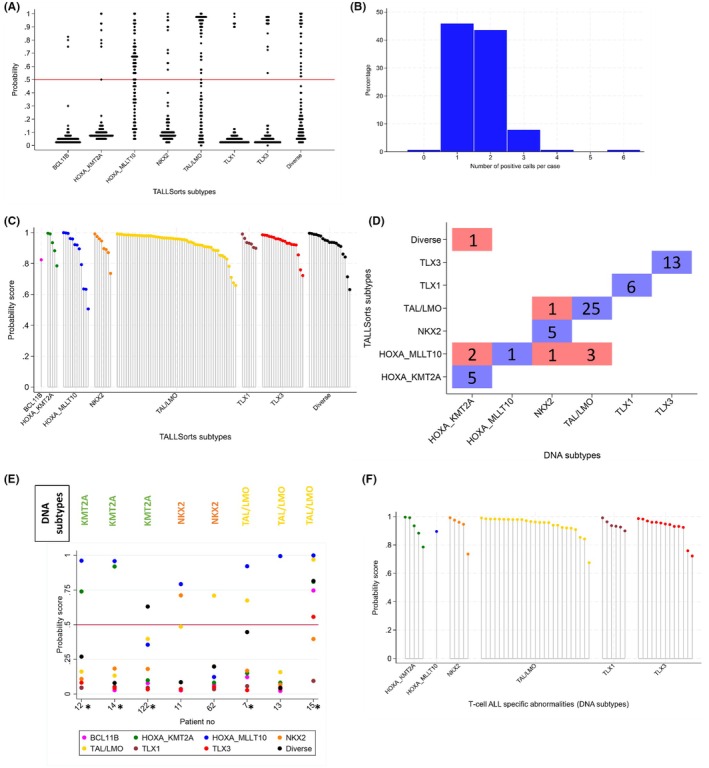
Overview of the results generated by the TALLSorts classifier across 126 patient samples. (A) shows the distribution of probability scores for all samples of all subtypes. The histogram in (B) shows the distribution of positive calls (>0.5) per sample. (C) shows the classification of 125 cases according to the highest probability score generated for that sample. (D) shows a confusion matrix for the 62 samples which could be assigned to a subtype by DNA testing. (E) shows all the probability scores for the eight cases from (D) which showed discrepant results (* indicates samples with poor quality RNA). (F) shows the highest probability scores for the 54 patients with concordant results between TALLSorts and the DNA testing.

Using the highest probability score for each sample, 125/126 (99%) cases were classified into one subtype (Figure 1C). The number of patients per subtype was *TAL/LMO* (*n* = 54, 43%), *TLX3* (*n* = 19, 15%), diverse (*n* = 19, 15%), *HOXA_MLLT10* (*n* = 12, 10%), *NKX2* (*n* = 8, 6%), *TLX1* (*n* = 7, 6%), *HOXA_KMT2A* (*n* = 5, 4%) and *BCL11B* (*n* = 1, 1%). The TALLSorts algorithm failed to classify one case (126) into any subtype despite good quality RNA. Traditional testing did not reveal any relevant fusions but Arriba detected a *KMT2A::MAML2* fusion (Table S1).

In the original TALLSorts study, the most frequent predicted subtype was *TAL/LMO* (46%) followed by *TLX3* (15%), diverse (15%), *TLX1* (7%), *NKX2* (7%), *HOXA_MLLT10* (4%), *HOXA_KMT2A* (3%) and *BCL11B* (1%), in agreement with the frequencies in our study, except for the *HOXA_MLLT10* subtype. *HOXA_MLLT10* was called twice as frequently in our study, tallying with our observation that this subtype had a diffuse distribution (Figure 1A), and accounted for most cases with >1 positive calls (Figure S1). The recent Children's Oncology Group (COG) study based on >1300 cases of T‐ALL reported RNA‐sequencing defined subtypes with frequencies similar to those found in this study: *TAL1* (DP) Double positive‐like (22%), (ETP) Early T‐cell precursor‐like (18%), *TAL1* αβ‐like (17%), *TLX3* (16%), *NKX2‐1* (6%) and *TLX1* (6%).^1^

Next, we compared the results of DNA‐based methods (cytogenetics, FISH and MLPA) with the TALLSorts subtypes in 63 cases where appropriate testing had been performed and found a concordance rate of 87% (55/63) (Figure 1D). Two of the discrepancies (cases 13, 14) were explained by fusions detected with Arriba. Case 13 had a *SIL::TAL1* rearrangement by MLPA and both a *SIL::TAL1* and *PICALM::MLLT10* rearrangement were called by Arriba but was only called as *HOXA_MLLT10* by TALLSorts (Figure 1E). Arriba revealed a *KMT2A::MLLT1* fusion in case 14 with the TALLSorts classifier generating probability scores of 0.92 and 0.96 for *HOXA_KMT2A* and *HOXA_MLLT10* subtypes respectively. No additional relevant fusions were detected in cases 7, 11, 12, 15, but in all four cases, TALLSorts called >1 subgroup with the second placed subtype matching the results of DNA testing (Figure 1E). We were not able to resolve the remaining two discrepancies (cases 62 and 122), but it is noteworthy that neither had high probability scores for the assigned subtype (both <0.75). In case 62 FISH detected a *NKX2* rearrangement but TALLSorts did not classify it in the *NKX2* subtype and no *NKX2* fusion was called by Arriba despite good quality RNA. Similarly, FISH detected a *KMT2A* fusion in both the diagnostic and relapse samples of case 122 which was not detected by Arriba and TALLSorts did not place it in the *HOXA_KMT2A* subtype. Factoring in the Arriba calls as well as the second subtype scores, the concordance rate would rise to 97% (61/63). Overall, 5/8 (63%) discrepant cases had poor RNA quality due to a high number of unmapped reads, compared to 11/54 (20%) concordant cases (*p* = 0.011). In addition, four of five samples with poor RNA quality had >1 call (Figure 1E). However, most poor quality samples (11/16, 69%) had concordant results, so they were not excluded from the study to provide a real‐life evaluation. Among 63 cases not assigned a TALLSort subtype by DNA, 11 had relevant fusions by RNA analysis Arriba which explained their classification and included two fusions missed by the DNA methods (Table S1).

Generally, our T‐ALL cohort had not been comprehensively tested for genetic abnormalities (Table S1). However, among 63 cases with T‐ALL specific abnormalities identified by DNA methods, 55 (95%) cases had probability scores of >0.65 corresponding to the correct TALLSorts subtype (Figure 1F). Among the five cases with TALLSorts probability scores of 0.65–0.80, one case had poor RNA quality, compared to 10/50 cases with probability scores of >0.80 further supporting our decision not to exclude these samples.

There were few differences between the seven subtypes in terms of clinical features and outcome (Table 1). However, patients in the *TAL/LMO* subtype had a higher white blood cell count (*p* = 0.004) and were more frequently MRD positive at the end of induction (*p* = 0.03). There was no difference in outcome between the subtypes. The same results were obtained even when we reassigned the five cases (7, 11, 12, 14 and 15) with aberrantly high *HOXA‐MLLT10* scores to relevant subtypes (Table S3). Finally, we examined the spectrum of secondary abnormalities by TALLSorts subtypes. The frequency of cases with *NOTCH1/FBXW7* mutations and *CDKN2A/B* deletion was high across most of the subtypes as reported by other studies. The exception was the diverse subtype, where only 2/9 (11%) had a *CDKN2A/B* deletion (Table S4).

**TABLE 1:** Patient characteristics and clinical outcomes of the subtypes detected by TALLSorts.

Variables	Values	Total no	*HOXA_KMT2A*	*HOXA_MLLT10*	*NKX2*	*TAL/LMO*	*TLX1*	*TLX3*	*Diverse*
Subtypes		124	5 (4%)	12 (10%)	8 (6%)	54 (44%)	7 (6%)	19 (15%)	19 (15%)
Sex	Male	96	3 (60%)	7 (58%)	6 (75%)	48 (89%)	5 (71%)	13 (68%)	14 (74%)
	Female	28	2 (40%)	5 (42%)	2 (25%)	6 (11%)	2 (29%)	6 (32)	5 (26%)
Age	Median (range)	124	10.0 (3.9–15.0)	9.8 (2.3–14.8)	4.1 (2.0–11.3)	8.6 (1.0–21.9)	7.5 (4.9–18.0)	6.9 (3.1–15.9)	9.0 (1.0–20.3)
WBC count, 10^9^/L	Median (range)	124	43.7 (8.6–269.0)	223.4 (11.0–777.0)	38.7 (10.6–102.5)	139.3 (9.5–881.0)	64.2 (8.0–414.0)	68.2 (11.8–393.7)	50.7 (1.0–522.3)
CNS involvement	Yes	9	0 (0%)	2 (17%)	0 (0%)	5 (10%)	0 (0%)	0 (0%)	2 (11%)
	No	112	4 (100)	10 (83%)	8 (100%)	47 (90%)	7 (100%)	19 (100%)	17 (89%)
MRD positive (≥0.01) at EOI	Yes	64	3 (75%)	4 (44%)	0 (0%)	35 (71%)	2 (29%)	9 (50%)	11 (100%)
	No	42	1 (25%)	5 (56%)	8 (100%)	14 (29%)	5 (71%)	9 (50%)	0 (0%)
Complete remission	Yes	122	5 (100%)	12 (100%)	8 (100%)	54 (100%)	7 (100%)	19 (100%)	17 (89%)
	No	2	0 (0%)	0 (0%)	0 (0%)	0 (0%)	0 (0%)	0 (0%)	2 (11%)
EFS at 5 years	% (95% CI)	124	80% (20–97)	75% (41–91)	88% (39–98)	78% (64–87)	71% (26–92)	95% (68–99)	66% (40–83)
RR at 5 years	% (95% CI)	122	20% (3–80)	18% (5–55)	13% (2–61)	19% (11–33)	29% (8–74)	5% (1–32)	29% (13–57)
OS at 5 years	% (95% CI)	124	100% (—)	75% (41–91)	100% (—)	81% (68–89)	100% (—)	100% (—)	71% (44–87)

*Note*: The single patient assigned to the *BCLL11B* subgroup, and the unclassified patient has not been included.

Abbreviations: BM, bone marrow; CNS, central nervous system; EFS, event‐free survival; EOI, end of induction; MRD, minimal/measurable residual disease; OS, overall survival; RR, relapse rate; WBC, white blood cell.

We have confirmed the utility of TALLSorts in an independent cohort. T‐ALL classification by traditional DNA tests is challenging due to the variety of mechanisms by which T‐cell oncogenes can be activated. Hence, TALLSorts offers a potential alternative to performing multiple tests. We observed a high concordance rate with Standard of care (SOC) techniques as well as the successful classification of cases with non‐standard rearrangements which were ambiguous or missed by SOC tests. However, TALLSorts does not detect all subtypes which is a limitation, but its creators plan the addition of new subtypes.^4^ The major limitation was the over‐calling of the *HOXA_MLLT10* subtype which accounted for >95% of cases with >1 positive calls. While we cannot exclude the possibility that these cases harbour a cryptic abnormality consistent with the *HOXA_MLLT10* subtype, this is unlikely for three reasons: (1) it was the only subtype to differ in frequency with the original TALLSorts study; (2) among the 12 cases called as *HOXA_MLLT10*, five cases produced valid second calls supported by DNA testing; and (3) the distribution of the calls was not discrete unlike in the original TALLSorts study. TALLSorts allows users to train custom models; such flexibility could enable its integration into a T‐ALL screening strategy.

## AUTHOR CONTRIBUTIONS

Conception and design: AVM, OG and CJH. Provision of data: TJ, DF, CS, AV, JR, PVV and MTR. Data analysis and interpretation: OG, REC, AE and AVM. Statistics: OG. Manuscript writing and final approval: all authors.

## CONFLICT OF INTEREST STATEMENT

TJ, DF and MTR were employees of Illumina, a public company that develops and markets systems for genetic analysis. All other authors declare no competing interests.

## Supporting information


**Figure S1.** The probability scores of each subtype predicted by TALLSorts across all cases.

**Table S1.** Demographic features, clinical features, sample detail, genetic subtype and TALLSorts data for all 126 cases in the study.

**Table S2.** Patient characteristics and clinical outcomes of tested and total (tested + non‐tested) cases with T‐ALL treated on UKALL2003 and UKALL2011.
**Table S3.** Characteristics and clinical outcomes of patients classified by the TALLSorts algorithm adjusting for cases with aberrantly high *HOXA_MLLT10* scores.
**Table S4.** Distribution of genetic alterations by TALLSorts predicted subtypes (number positive/number tested).
