# Supplementary material for: Benchmarking the paediatric T‐cell ALL subtype classifier, TALLSorts
Source: Br J Haematol. 2025 Dec 12;208(2):732–6. doi: 10.1111/bjh.70263 (PMC12916182; doi:10.1111/bjh.70263)
Supplement: Supplementary file 1 — Figure S1. The probability scores of each subtype predicted by TALLSorts across all cases. [file BJH-208-732-s001.docx]

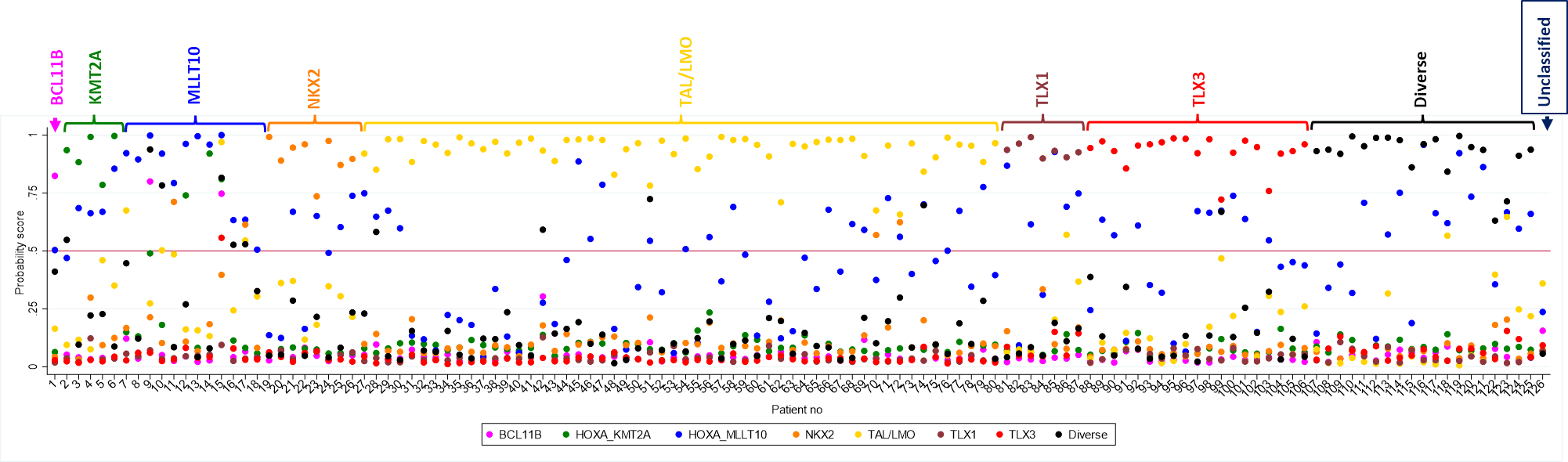


**Figure S1.** The probability scores of each subtype predicted by TALLSorts across all cases. Subtypes are represented by different colours.
