# Supplementary material for: Benchmarking the paediatric T‐cell ALL subtype classifier, TALLSorts
Source: Br J Haematol. 2025 Dec 12;208(2):732–6. doi: 10.1111/bjh.70263 (PMC12916182; doi:10.1111/bjh.70263)
Supplement: Supplementary file 3 — Table S2. Patient characteristics and clinical outcomes of tested and total (tested + non‐tested) cases with T‐ALL treated on UKALL2003 and UKALL2011. Table S3. Characteristics and clinical outcomes of patients classified by the TALLSorts algorithm adjusting for cases with aberrantly high HOXA_MLLT10 scores. Table S4. Distribution of genetic alterations by TALLSorts predicted subtypes (number positive/number tested). [file BJH-208-732-s002.docx]

Supplementary Information for “**Benchmarking the paediatric T-cell ALL subtype classifier, TALLSorts”**

Ozcan Gulbey^1^, Terena James^2^, Ruth E Cranston^1^, Dagmara Furmanczyk^2^, Claire Schwab^1^, Anna Lawson^4^, Pam Kearns^4^, Ajay Vora^3^, Juliette Roels^4^, Pieter Van Vlierberghe^4^, Christine J Harrison^1^, Mark T Ross^2^, Amir Enshaei^1^, Frederik W van Delft^1^, Anthony V Moorman^1^

^1^Leukaemia Research Cytogenomics Group, Centre for Cancer, Translational and Clinical Research Institute, Newcastle University, Newcastle upon Tyne, UK; ^2^Illumina Cambridge Ltd., Granta Park, Great Abington, Cambridge, UK; ^3^Cancer Research UK Clinical Trials Unit, School of Medical Sciences, College of Medicine and Health, University of Birmingham, B15 2TT. ^4^Department of Haematology, Great Ormond Street Hospital, London, UK; ^5^Department of Biomolecular Medicine, Ghent University, Ghent, Belgium.

**Methods**

***RNA-sequencing***

RNA-sequencing (RNA-seq) was performed in two batches in separate laboratories. RNA was extracted from bone marrow samples taken at initial diagnosis.

Batch A (n=29): RNA quality was estimated using the Agilent 2100 bioanalyzer (Agilent Technologies). Paired end, poly(A) enriched, stranded RNA-seq was performed with the True Seq Stranded mRNA Sample Preparation Kit (Illumina). Libraries were sequenced on a NextSeq500 sequencer (Illumina) with a read length of 75 base-pairs. RNA-seq reads were aligned and quantified using GRCh38 and Gencode v.24 and STAR v.2.4.2a (1) with the parameter quantMode GeneCounts.

Batch B (n=97): The quality of RNA was assessed using the Agilent 4200 Tapestation System, using High Sensitivity tapes. The concentration was assessed using the Gemini^TM^ XPS Microplate Spectroflurometer from Molecular Devices and the Quant-iT HS RNA assay. Libraries were prepared using the Illumina Stranded Total RNA Prep, Ligation with Ribo-Zero Plus, with additional custom depletion probes, using 100-400ng RNA. Libraries were sequenced on a NovaSeq 6000 system using 101 base paired-end chemistry.

Trimmed FASTQ reads were aligned to hg38 and Gencode 28 using STAR (2.7.0e) (1) and Arriba (v1.2.0) (2), for the detection of gene fusions.

The blast percentage of these samples was available for 87 patients (Table S1) and was 80% or more in 79 (91%) cases. There was no difference in the average blast percentage between the 72 samples with good RNA quality and the 15 samples with poor RNA quality (88% v 86%, *p*=NS) (Table S1).

***Statistical Analysis***

Comparison tests (Chi-square, Fisher exact, or Mann-Whitney U) were used to check if differences between the groups of interest were statistically significant. Log-rank test was performed for the following time-to-event analyses until the date of last contact: event-free survival (from diagnosis to relapse, second tumour or death), relapse rate for patients with complete remission (from diagnosis to relapse), and overall survival (from diagnosis to death). The STATA (v.16) was used for the statistical analyses with the significance level of 0.05, and *p*-values (*p*) were two-sided in all analyses.

**Reference for the supplementary document**

1. Dobin A, Davis CA, Schlesinger F, Drenkow J, Zaleski C, Jha S, Batut P, Chaisson M, Gingeras TR. STAR: ultrafast universal RNA-seq aligner. Bioinformatics. 2013 Jan 1;29(1):15-21. doi: 10.1093/bioinformatics/bts635. Epub 2012 Oct 25.

2. Uhrig S, Ellermann J, Walther T, Burkhardt P, Fröhlich M, Hutter B, Toprak UH, Neumann O, Stenzinger A, Scholl C, Fröhling S, Brors B. Accurate and efficient detection of gene fusions from RNA sequencing data. Genome Research 2021 Mar; 448-460.

**Figure Legends:**

**Figure S1**. The probability scores of each subtype predicted by TALLSorts across all cases. Subtypes are represented by different colours.

**Table S2:** Patient characteristics and clinical outcomes of tested and total (tested + non-tested) cases with T-ALL treated on UKALL2003 and UKALL2011.

| **Variables** | **Values** | **Total no** | **Tested cohort** | **Non-tested cohort** | ***p*-value** |
| --- | --- | --- | --- | --- | --- |
| Cohorts |  | 833 | 126 (15%) | 707 (85%) | - |
| Sex | Male | 627 | 97 (77%) | 530 (75%) | 0.63 |
|  | Female | 206 | 29 (23%) | 177 (25%) |  |
| Age, years | Median (range) | 833 | 8.8 (1.0-21.9) | 10.0 (1.0-24.3) | **0.005** |
| WBC count, 10^9^/L | Median (range) | 830 | 108.9 (1.0-881.0) | 85.2 (0.4-1800.0) | 0.16 |
| CNS involvement | Yes | 46 | 9 (7%) | 37 (6%) | 0.43 |
|  | No | 748 | 114 (93%) | 634 (94) |  |
| BM blast at diagnosis | Median (range) | 473 | 91% (14-100) | 90% (4-100) | **0.03** |
| BM blast at day 8 | Median (range) | 729 | 14% (0-96) | 10% (0-99) | 0.09 |
| BM blast at day 28 | Median (range) | 734 | 1% (0-70) | 1% (0-95) | 0.91 |
| MRD positive (≥0.01) at EOI | Yes | 381 | 65 (61%) | 316 (59%) | 0.75 |
|  | No | 261 | 42 (39%) | 219 (41%) |  |
| Complete remission | Yes | 788 | 124 (98%) | 664 (94%) | **0.04** |
|  | No | 44 | 2 (2%) | 42 (6%) |  |
| EFS at 5 years | % (95% CI) | 833 | 78% (70-85) | 79% (76-82) | 0.85 |
| RR at 5 years | % (95% CI) | 788 | 18% (12-26) | 16% (12-19) | 0.48 |
| OS at 5 years | % (95% CI) | 833 | 85% (78-91) | 86% (83-88) | 0.89 |

Abbreviations: WBC, white blood cell; CNS, central nervous system; BM, bone marrow; MRD, minimal/measurable residual disease; EOI, end of induction; EFS, event-free survival; RR, relapse rate; OS, overall survival.

**Table S3:** Characteristics and clinical outcomes of patients classified by the TALLSorts algorithm adjusting for cases with aberrantly high *HOXA_MLLT10* scores. Five cases (7, 11, 12, 14 and 15) were re-assigned based on their second highest probability scores, and two patients have been excluded from this table; the single patient in *BCLL11B* subtype and the case which was not classified by the classifier.

| **Variables** | **Values** | **Total no** | ***HOXA_KMT2A*** | ***HOXA_MLLT10*** | ***NKX2*** | ***TAL/LMO*** | ***TLX1*** | ***TLX3*** | **Diverse** |
| --- | --- | --- | --- | --- | --- | --- | --- | --- | --- |
| Total number |  | 124 | 7 (6%) | 7 (6%) | 9 (7%) | 56 (45%) | 7 (6%) | 19 (15%) | 19 (15%) |
| Sex | Male | 96 | 4 (57%) | 5 (71%) | 6 (67%) | 49 (88%) | 5 (71%) | 13 (68%) | 14 (74%) |
|  | Female | 28 | 3 (43%) | 2 (29%) | 3 (33%) | 7 (12%) | 2 (29%) | 6 (32%) | 5 (26%) |
| Age, years | Median (age) | 124 | 10.0 (3.9-15.0) | 9.6 (4.1-13.2) | 4.0 (2.0-11.3) | 8.8 (1.0-21.9) | 7.5 (4.9-18.0) | 6.9 (3.1-15.9) | 9.0 (1.0-20.3) |
| WBC count, 10^9^/L | Median (age) | 124 | 43.7 (8.6-269.0) | 59.0 (11.0-777.0) | 55.4 (10.6-366.0) | 148.0 (9.5-881.0) | 64.2 (8.0-414.0) | 68.2 (11.8-393.7) | 50.7 (1.0-522.3) |
| CNS involvement | Yes | 9 | 0 (0%) | 1 (14%) | 0 (0%) | 6 (11%) | 0 (0%) | 0 (0%) | 2 (11%) |
|  | No | 112 | 6 (100%) | 6 (86%) | 9 (100%) | 48 (89%) | 7 (100%) | 19 (100%) | 17 (89%) |
| MRD positive (≥0.01) at EOI | Yes | 64 | 4 (67%) | 2 (40%) | 1 (11%) | 35 (70%) | 2 (29%) | 9 (50%) | 11 (100%) |
|  | No | 42 | 2 (33%) | 3 (60%) | 8 (89%) | 15 (30%) | 5 (71%) | 9 (50%) | 0 (0%) |
| Complete remission | Yes | 122 | 7 (100%) | 7 (100%) | 9 (100%) | 56 (100%) | 7 (100%) | 19 (100%) | 17 (89%) |
|  | No | 2 | 0 (0%) | 0 (0%) | 0 (0%) | 0 (0%) | 0 (0%) | 0 (0%) | 2 (11%) |
| EFS at 5 years | % (95% CI) | 124 | 71% (26-92) | 86% (33-98) | 89% (43-98) | 77% (63-86) | 71% (26-92) | 95% (68-99) | 66% (40-83) |
| RR at 5 years | % (95% CI) | 122 | 17% (3-73) | 14% (2-67) | 11% (2-57) | 20% (12-34) | 29% (8-74) | 5% (1-32) | 29% (13-57) |
| OS at 5 years | % (95% CI) | 124 | 86% (33-98) | 86% (33-98) | 100% (-) | 80% (67-88) | 100% (-) | 100% (-) | 71% (44-87) |

Abbreviations: WBC, white blood cell; CNS, central nervous system; MRD, minimal/measurable residual disease; EOI, end of induction; EFS, event-free survival; RR, relapse rate; OS, overall survival.

**Table S4:** Distribution of genetic alterations by TALLSorts predicted subtypes (number positive / number tested).

| **Abnormalities** | ***HOXA_KMT2A*** | ***HOXA_MLLT10*** | ***NKX2*** | ***TAL/LMO*** | ***TLX1*** | ***TLX3*** | **Diverse** |
| --- | --- | --- | --- | --- | --- | --- | --- |
| Subtypes | 5 (4%) | 12 (10%) | 8 (6%) | 54 (44%) | 7 (6%) | 19 (15%) | 19 (15%) |
| *NOTCH1* mutations | 0/2 (0%) | 5/6 (83%) | 6/6 (100%) | 16/30 (53%) | 4/4 (100%) | 10/14 (71%) | 4/6 (67%) |
| FBXW7 mutations | 1/2 (50%) | 0/6 (0%) | 1/5 (20%) | 2/29 (7%) | 0/4 (0%) | 3/14 (21%) | 1/6 (17%) |
| *PHF6* mutations | - | 0/6 (0%) | 2/6 (33%) | 2/18 (11%) | 1/3 (33%) | 4/11 (36%) | 1/4 (25%) |
| *PTEN* mutations | 0/2 (0%) | 1/6 (17%) | 1/6 (17%) | 5/29 (17%) | 0/4 (0%) | 0/14 (0%) | 0/6 (0%) |
| *WT1* mutations | - | 1/6 (17%) | 1/6 (17%) | 0/18 (0%) | 0/3 (0%) | 1/11 (9%) | 1/4 (25%) |
| *IL7R* mutations | - | 0/6 (0%) | 1/6 (17%) | 1/18 (6%) | 0/3 (0%) | 1/11 (9%) | 0/4 (0%) |
| *JAK1/3* mutations | - | 1/6 (17%) | 0/6 (0%) | 0/18 (0%) | 0/3 (0%) | 2/11 (18%) | 2/4 (50%) |
| *N/K RAS* mutations | 0/2 (0%) | 0/4 (0%) | 0/3 (0%) | 1/22 (5%) | 0/2 (0%) | 1/9 (11%) | 2/3 (67%) |
| *PTEN* deletions | 0/3 (0%) | 1/9 (11%) | 0/7 (0%) | 4/47 (9%) | 0/7 (0%) | 2/18 (11%) | 0/12 (0%) |
| *LEF1* deletions | 0/3 (0%) | 1/9 (11%) | 2/7 (29%) | 3/47 (6%) | 0/7 (0%) | 0/17 (0%) | 0/12 (0%) |
| *IKAROS* deletions | 0/2 (0%) | 1/5 (20%) | 0/2 (0%) | 0/21 (0%) | 0/2 (0%) | 1/6 (17%) | 1/9 (11%) |
| *CDKN2A/B* deletions | 1/5 (20%) | 11/12 (92%) | 6/8 (75%) | 44/54 (81%) | 7/7 (100%) | 16/18 (89%) | 2/19 (11%) |
